# Reusable BiOI-Modified CuWO_4_ Heterojunction Films and Their Excellent Photocatalytic Oxidation Activity of Nanoplastics and Methylene Blue

**DOI:** 10.3390/nano15201579

**Published:** 2025-10-16

**Authors:** Te Hu, Liang Hao, Xiaohui Zhao, Sujun Guan, Yun Lu

**Affiliations:** 1 School of Materials and Energy, Foshan University, Foshan 528000, China; hute98@hotmail.com; 2 School of Mechanical Engineering, Tianjin University of Science and Technology, Tianjin 300457, China; zhaoxiaohui2281016@mail.tust.edu.cn; 3 School of Physics, Henan University of Technology, Zhengzhou 450001, China; guansujun@haut.edu.cn; 4 School of Intelligent Manufacturing, Chengdu Technological University, Chengdu 611730, China; 5 Graduate School of Science and Engineering, Chiba University, Chiba 263-8522, Japan

**Keywords:** BiOI/CuWO_4_, heterojunction, films, photocatalytic degradation, nanoplastics, organic dye

## Abstract

CuWO_4_ films were prepared on FTO glass substrates by the hydrothermal method. To improve their photocatalytic activity, the CuWO_4_ films were further modified with BiOI using the successive ionic layer adsorption and reaction (SILAR) method. Characterization results indicate that BiOI and CuWO_4_ achieved nanoscale mixing and formed a Type II *p*-*n* heterojunction. The heterojunction formation not only extends the light absorption threshold of CuWO_4_ from 530 nm to 660 nm but also enhances the light absorption capacity across the entire solar spectrum. More importantly, the heterojunction formation facilitates the separation and transfer of photogenerated carriers and inhibits the recombination of photogenerated electrons and holes, which is evidenced by the results of PL spectra, photocurrent density, and EIS spectra. Compared with individual CuWO_4_ films, the photocatalytic activity of BiOI/CuWO_4_ heterojunction films in degrading the organic dye MB is increased by up to 1.17 times. Additionally, BiOI/CuWO_4_ heterojunction films exhibit certain activity in the photocatalytic degradation of polystyrene (PS) nanoplastics and are capable of reducing the average particle size of nanoplastics from 425 nm to 325 nm within 80 h.

## 1. Introduction

Copper tungstate (CuWO_4_), a typical indirect *n*-type semiconductor, has garnered increasing attention in recent years as a promising photocatalyst [1,2,3]. It possesses numerous advantages as a high-performance photocatalyst, including a moderate bandgap, excellent chemical stability, low toxicity, and high light-to-current conversion ability [4,5,6,7]. Notably, it exhibits a bandgap ranging from 2.2 eV to 2.4 eV, enabling strong absorption and efficient utilization of visible light, which accounts for over 40% of the total energy in solar radiation spectra [8,9]. However, the inherently low charge carrier mobility and rapid bulk recombination of photogenerated electron–hole pairs pose significant challenges that substantially compromise its photocatalytic performance [10,11,12]. Researchers have explored numerous strategies to address these challenges and enhance photocatalytic activity, including elemental doping [13,14], noble metal modification [15,16], defect engineering [17,18,19], surface sensitization [20,21], and heterostructure construction [22,23,24,25,26]. In particular, the strategy of constructing heterostructures has gained the most widespread application due to its convenient preparation methods, low cost, and remarkable effectiveness. Specifically, constructing Type II, Z-scheme, and S-scheme heterojunctions proves most effective in enhancing the photocatalytic activity of CuWO4 [27]. Semiconductor materials used for constructing heterojunctions include WO_3_ [15], CuO [23], ZnO [28], TiO_2_ [2], C_3_N_4_ [29,30], Bi_2_S_3_ [31,32], VO_2_ [22], Co_3_O_4_ [33], Bi_2_MoO_6_ [34], Ag_3_PO_4_ [35], ZnFe_2_O_4_ [36], BiOCl [3], and BiOI [8,37], among others.

Among these semiconductors, BiOI stands out as particularly remarkable. The narrow bandgap of BiOI (1.7–2.3 eV) extends the visible light absorption range of the composite beyond that of CuWO_4_ [38]. Crucially, their well-matched band structures facilitate the construction of a Type II heterojunction. This configuration promotes the spatial separation of photogenerated carriers: electrons migrate toward the CuWO_4_ conduction band while holes transfer to the BiOI valence band. The resultant suppression of charge recombination significantly improves the utilization efficiency of photogenerated carriers for surface redox reactions, thereby boosting the overall photocatalytic performance of CuWO_4_. In our published work, we prepared CuWO_4_ on a copper mesh substrate via anodic oxidation and subsequently formed BiOI on the CuWO_4_ surface using the successive ionic layer adsorption and reaction (SILAR) method to construct a heterojunction between them. However, both CuWO_4_ and BiOI tend to peel off from the substrate, leading to a rapid decline in the photocatalytic activity of this heterojunction film and, consequently, limiting its application [37].

In this study, FTO glass plates were used as the substrates to prepare BiOI-modified CuWO_4_ films via a hydrothermal method combined with the SILAR technique. The prepared films exhibit excellent photocatalytic activity in photocatalytic degradation of both organic dyes and nanoplastics. Notably, neither BiOI nor CuWO_4_ detach during operation, demonstrating robust interfacial adhesion strength. This heterojunction films hold significant promise for application in water remediation technologies, specifically for the treatment of organic dye pollution and nanoplastic contamination.

## 2. Materials and Methods

### 2.1. Preparation of CuWO_4_ Films

First, dissolve 0.29 g of CuCl_2_ and 0.42 g of ammonium metatungstate hydrate ((NH_4_)_6_H_2_W_12_O_40_·3H_2_O) (molar ratio: 15.65) in a mixed solvent of ethylene glycol (15 mL) and deionized water (30 mL). Stir vigorously until complete dissolution is achieved. Second, transfer the homogeneous solution to a 60 mL polytetrafluoroethylene (PTFE) liner. Insert an FTO conductive glass substrate (sheet resistance ≤ 14 Ω/sq), pre-cleaned by sequential ultrasonication in acetone, ethanol, and deionized water, into the liner with its conductive side facing upward. Subsequently, seal the autoclave and place it in a muffle furnace. Heat to 160 °C at a ramping rate of 10 °C/min and maintain this temperature for 4 h. Allow the autoclave to cool naturally to room temperature inside the furnace. After that, retrieve the sample and rinse it alternately with deionized water and absolute ethanol three times under gentle agitation to remove residual reactants. Finally, anneal the sample in the muffle furnace by heating to 500 °C at 10 °C/min, holding for 2 h, then cooling naturally in the furnace to room temperature. The prepared sample is designated as CuWO_4_.

### 2.2. Preparation of BiOI/CuWO_4_ Heterojunction Films

First, immerse the prepared CuWO_4_ sample in a 0.08 mol/L bismuth nitrate ethylene glycol solution for 10 s to allow sufficient adsorption of Bi^3+^ ions on the film’s surface. Then, remove the sample and let it dry in air for 10 min to ensure complete adsorption. Next, transfer the sample into a 0.08 mol/L KI aqueous solution and soak it for 10 s to allow I^−^ ions to react with the adsorbed Bi^3+^, forming BiOI. Afterward, remove the sample and let it dry again for 10 min. These steps constitute one complete SILAR cycle. After repeating this cycle a set number of times, place the sample in a vacuum drying oven and heat-treat it at 60 °C for 4 h. The resulting sample is designated as BiOI/CuWO_4_. All chemical reagents used are of analytical grade and employed without further purification.

### 2.3. Characterization

The phase composition of the samples was analyzed using a Bruker D8 Advance X-ray diffractometer (XRD) with a Cu Kα radiation source (λ = 1.5406 Å). The operating voltage and current were set at 40 kV and 40 mA, respectively. The scanning range was 20° to 80° (2θ) with a scan rate of 10°/min. The surface morphology and elemental distribution of the samples were characterized by field-emission scanning electron microscopy (FE-SEM) (Apreo, FEI, Hillsboro, OR, USA). The acceleration voltage and beam current were set at 2 kV and 25 pA, respectively. The microstructure of the samples was characterized by transmission electron microscopy (TEM) using a Talos G2 200X system (FEI, Hillsboro, OR, USA), operated at an accelerating voltage of 120 kV. The elemental composition, oxidation states, and surface chemical states of the samples were analyzed using X-ray photoelectron spectroscopy (XPS) on a Escalab 250Xi spectrometer (Thermo Fisher Scientific, Waltham, MA, USA). Charge correction was applied using the C 1s peak (284.5 eV) to ensure data accuracy. The optical response properties of the samples were systematically characterized using a UV-3600Plus UV–vis spectrophotometer (Shimadzu, Kyoto, Japan) with full-spectrum scanning in the wavelength range of 200–800 nm. The charge transfer efficiency at the heterojunction interfaces and the defect concentration within the materials were qualitatively analyzed using a FLS 1000/FS5 fluorescence spectrometer (Edinburgh Instruments, Edinburgh, UK). A 325 nm laser source was employed as the excitation wavelength. The mineralization of PS nanoplastics was evaluated by measuring total organic carbon (TOC) using a Shimadzu TOC-L analyzer (Shimadzu, Kyoto, Japan).

The photoelectrochemical properties of the samples were systematically investigated using a CHI 660E electrochemical workstation (CH Instruments, Shanghai, China) equipped with a 300 W xenon lamp light source. Measurements were carried out in a standard three-electrode configuration, where the as-prepared sample served as the working electrode, a platinum sheet as the counter electrode, and a saturated calomel electrode as the reference electrode. All electrochemical tests were performed in 0.1 M Na_2_SO_4_ aqueous solution as the supporting electrolyte.

### 2.4. Photocatalytic Activity Evaluation

The photocatalytic activity evaluation is carried out as follows. First, immerse the prepared sample in a 50 mL glass beaker containing 20 mL of a methylene blue (MB) aqueous solution (10 mg/L). Prior to light irradiation, the system is kept in dark conditions for 30 min to establish adsorption–desorption equilibrium between the MB molecules and catalyst surface, thereby eliminating potential interference from physical adsorption during photocatalytic activity evaluation. The reaction system is irradiated using a 300 W xenon lamp equipped with an AM 1.5 G filter to simulate solar light irradiation, with the incident light intensity maintained at 45 mW/cm^2^ at the sample’s surface. The temperature of the reaction system is controlled by a circulating water cooling system throughout the evaluation. In total, 3 mL of the reaction solution is collected at 30 min intervals for UV–vis spectrophotometric analysis and immediately returned to the reactor after measurement to maintain a constant reaction volume. A parallel blank control experiment is performed under identical conditions to account for the self-degradation of MB under light irradiation. The degradation activity is calculated on the basis of the variation in the MB concentration using UV–vis spectroscopy.

The photocatalytic degradation experiment for nanoplastics is performed according to the following procedure. A 2 g/L polystyrene (PS) suspension is prepared using PS nanospheres with an average diameter of 425 nm. The prepared sample is immersed in a 50 mL glass beaker containing the PS suspension. The photocatalytic reaction is conducted under identical illumination conditions to the MB degradation experiment. After the evaluation, the sample is transferred to a clean beaker containing 50 mL ethanol and subjected to ultrasonic treatment to completely detach the residual PS nanospheres from the catalyst surface. The resulting suspension is drop-cast onto silicon wafers, and after ethanol evaporation, the size distribution of the nanospheres is analyzed by SEM. Fourier transform infrared spectroscopy (FTIR) is employed to evaluate the photocatalytic activity in the 400–4000 cm^−1^ spectral range.

## 3. Results and Discussion

**Figure 1:**
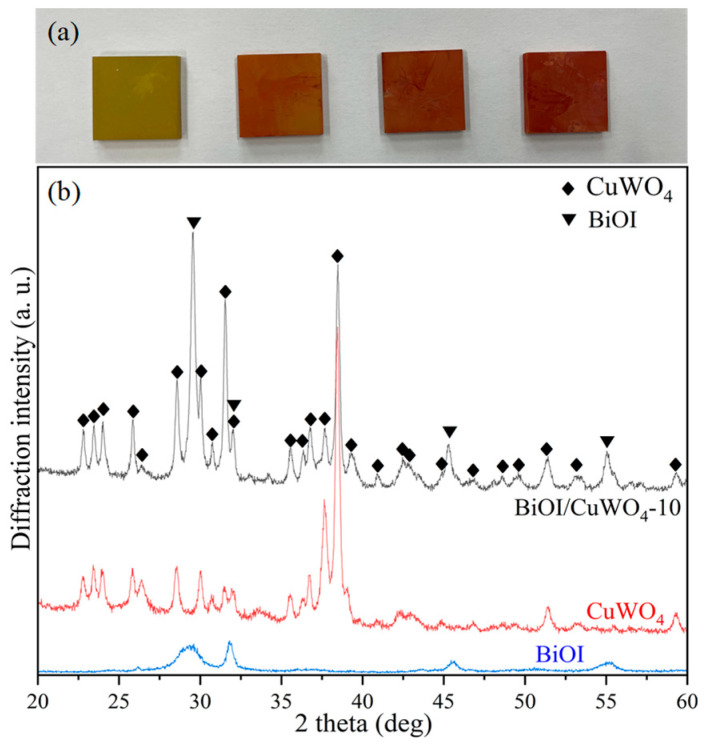
(**a**) Appearance and (**b**) XRD patterns of the prepared samples of CuWO_4_ and BiOI/CuWO_4_-10.

Figure 1a shows a photo of the appearance of the prepared samples. From left to right, they are CuWO_4_, BiOI/CuWO_4_-5, BiOI/CuWO_4_-10, and BiOI/CuWO_4_-20. The color of the CuWO_4_ sample is yellowish-green, which is consistent with the color of CuWO_4_ reported in the literature [24,39,40]. After the SILAR operation, the surface of the FTO glass turns a brick-red color, and the color gradually deepens with the increase in the number of SILAR cycles. This color is consistent with the color of BiOI reported in the literature [41,42,43]. Figure 1b shows the XRD patterns of the CuWO_4_, BiOI, and BiOI/CuWO_4_-10 samples. For the CuWO_4_ sample, the detected diffraction peaks correspond to the triclinic CuWO_4_ indexed to the standard diffraction database (JCPDS No. 72-0616). This indicates that CuWO_4_ crystals were formed during the hydrothermal process and the subsequent annealing process. For the BiOI sample, four distinct diffraction peaks appear at 2θ = 29.7°, 31.7°, 45.5°, and 55.3°, which correspond to the (012), (110), (020), and (122) crystal planes of tetragonal BiOI, respectively (JCPDS No. 73-2062). In the BiOI/CuWO_4_-10 sample, the abovementioned diffraction peaks of CuWO_4_ and BiOI are observed, which indicates the formation of BiOI/CuWO_4_ composite films.

**Figure 2:**
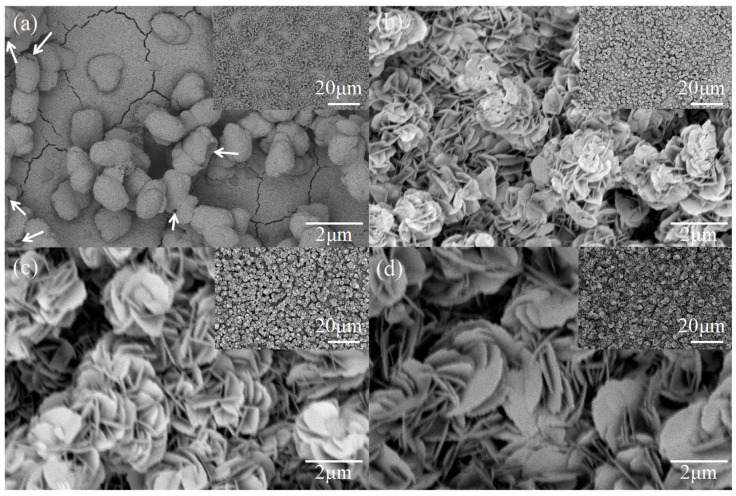
SEM images of the prepared samples: (**a**) CuWO_4_, (**b**) BiOI-CuWO_4_-5, (**c**) BiOI-CuWO_4_-10, and (**d**) BiOI-CuWO_4_-20.

Figure 2 shows the SEM images of the prepared CuWO_4_ and BiOI/CuWO_4_ samples. In Figure 2a, hierarchical hollow spherical particles with a particle size of approximately 1 μm have adhered to the surface of the porous film. This structure has been characterized in published works on CuWO_4_ photocatalyst [8,26,44]. After the SILAR process, numerous circular flake-like products with a thickness of several tens of nanometers and a diameter of approximately 1 micrometer formed on the surface of the CuWO_4_ film. Dozens of such flake-like products assemble together to form flower-like structures with a diameter of approximately 2 μm. As the number of SILAR cycles increases from 5 to 20, the quantity of these micrometer-scale flower-like structures continues to grow, eventually completely covering the CuWO_4_ film. It is speculated that this micrometer-scale flower-like product is BiOI, which is a typical morphology of BiOI [38]. In the following, other detection methods will be used to further confirm the products.

**Figure 3:**
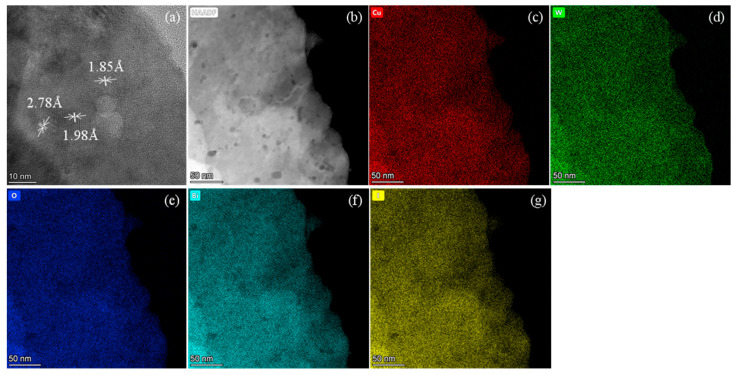
(**a**) HRTEM image, (**b**) HADDF image, and (**c**–**g**) element mappings of the BiOI/CuWO_4_-10 sample.

After ultrasonic cleaning of the prepared BiOI/CuWO_4_-10, the supernatant was taken and dropped onto a copper grid to prepare the TEM sample. In Figure 3a, some interplanar spacings were measured to be 2.78 and 1.85 Å, corresponding to the $\left(1\overset{-}{1}1\right)$ and (130) crystal planes of CuWO_4_, respectively. The interplanar spacing of 1.98 Å corresponds to the (014) plane of BiOI. In Figure 3b, different contrasts in HAADF mode indicate the coupling of BiOI and CuWO_4_. Meanwhile, elemental mapping was conducted, as shown in Figure 3c–g. The elements Cu, W, O, Bi, and I are uniformly distributed throughout the scanned area. Notably, some particles exhibit lower intensities of both Bi and I (Figure 3f,g), suggesting the presence of CuWO_4_ in these regions. This observation aligns with the general principle that lighter elements appear darker in HAADF imaging.

**Figure 4:**
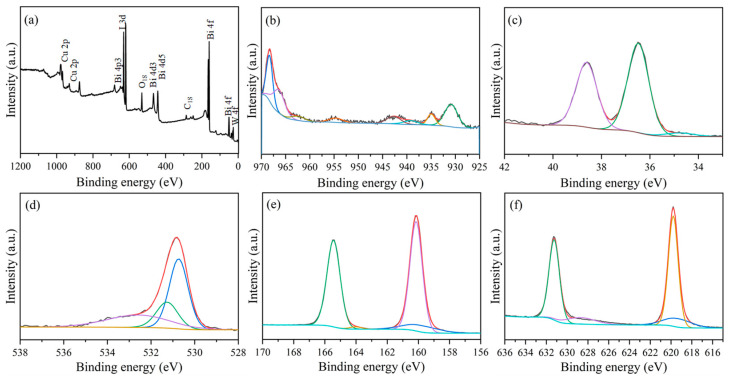
XPS spectra of the BiOI/CuWO_4_-10 sample: (**a**) survey spectrum, (**b**) Cu 2p, (**c**) W 4f, (**d**) O 1s, (**e**) Bi 4f, and (**f**) I 3d.

Figure 4a illustrates the XPS survey spectrum of the prepared BiOI/CuWO_4_-10 sample. It can be observed that the sample contains six elements, namely Cu, W, O, Bi, I, and C. The C element should be attributed to CO_2_ adsorbed onto the sample from the atmosphere, as well as adventitious carbon [45]. The high-resolution XPS spectrum of Cu 2p is shown in Figure 4b. The binding energies at 935.0 and 954.8 eV belong to Cu^2+^ in the lattice sites of CuWO_4_ [8,44,45]. For the element W (Figure 4c), two XPS peaks at the binding energies of 36.5 and 38.7 eV were detected. They should match with the W 4f_7/2_ and W 4f_5/2_ of W^6+^ in CuWO_4_ [46]. In the XPS spectrum of O 1s (Figure 4d), three peaks at the binding energies of 530.8, 531.2, and 532.7 eV should derive from the presence of W-O, Cu-O, and surface hydroxide adsorbed on the sample [47,48]. For the element Bi (Figure 4e), two peaks at the binding energies of 160.2 and 165.5 eV were detected. They should be attributed to the Bi 4f_7/2_ and Bi 4f_5/2_ of Bi^3+^ ions in BiOI [41,49]. In the XPS spectrum of I 3d (Figure 4f), two peaks at the binding energies of 619.8 and 631.2 eV should correspond to the I 3d_3/2_ and I 3d_5/2_ of I^−^ ions in BiOI [49,50,51].

**Figure 5:**
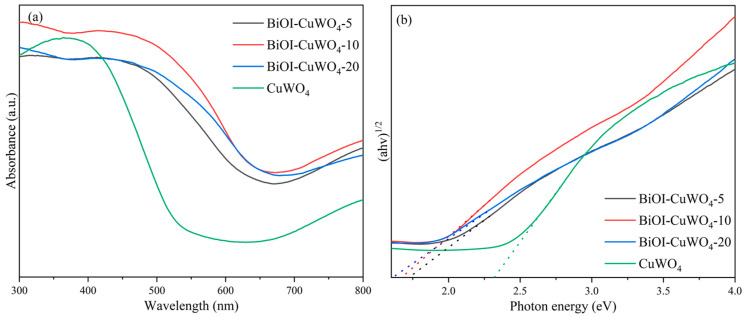
Optical properties of the prepared samples: (**a**) UV–vis diffuse reflection spectra, and (**b**) Tauc plots.

Figure 5 shows the optical absorption spectra of the prepared CuWO_4_ and BiOI/CuWO_4_ films. The individual CuWO_4_ films exhibit an intense absorption capacity with an absorption threshold range of less than 530 nm. For the BiOI/CuWO_4_ heterojunction films, they exhibit enhanced light absorption capacity over the entire ultraviolet–visible–near-infrared wavelength range, with the absorption threshold extending to approximately 660 nm. Among them, the light absorption capacity of the BiOI/CuWO_4_-10 sample is the strongest among all prepared samples. The Tauc plots of the prepared CuWO_4_ and BiOI/CuWO_4_ films are illustrated in Figure 5b. The bandgap values can be estimated by extrapolating the linear region of the Tauc plots to the x-axis ((*αhν*)^1/2^ = 0) [9]. The calculated bandgap value of the pristine CuWO_4_ films is 2.33 eV, which is consistent with the absorption threshold of CuWO_4_ at 530 nm. On the other hand, the bandgap values of the BiOI/CuWO_4_ heterojunction films decreased to varying degrees. Specifically, as the number of SILAR cycles increased from 5 to 20, the bandgap values of the samples decreased from 1.74 eV to 1.55 eV. The reduction in the bandgap values of the BiOI/CuWO_4_ heterojunction films is attributed to the bandgap value of BiOI being much smaller than that of CuWO_4_. Consequently, increasing the BiOI loading gradually reduces the bandgap. Moreover, the bandgap reduction enhances the absorption and utilization of visible light and even near-infrared light in sunlight by the photocatalyst.

**Figure 6:**
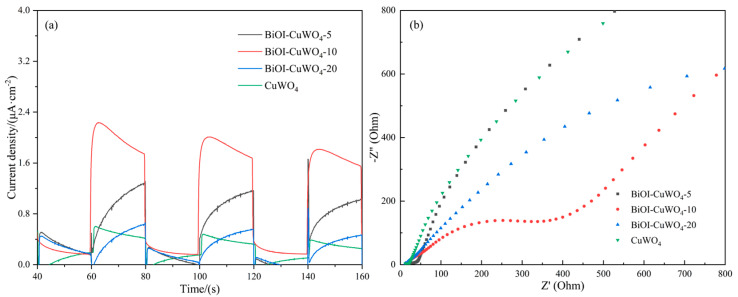
(**a**) Photocurrent density and (**b**) EIS of the prepared samples.

Photocurrent density reflects the light absorption capacity, carrier generation efficiency, separation efficiency, migration performance, surface reaction activity, and conductivity of photocatalytic materials. Under identical conditions, a higher photocurrent density indicates higher photocatalytic activity. Thus, photocurrent density measurement is widely employed to evaluate the photocatalytic activity [3,20,39]. Figure 6a depicts the photocurrent response of the prepared samples. It can be observed that the photocurrent density of the single CuWO_4_ films is approximately 0.4 μA/cm^2^ under simulated sunlight irradiation. After coupling with BiOI, the photocurrent densities of the BiOI/CuWO_4_ heterojunction films all increase to varying degrees. In particular, the photocurrent density of the BiOI/CuWO_4_-10 sample reaches a maximum of 2.2 μA/cm^2^, which is 5.5 times that of the CuWO_4_ films. Electrochemical impedance spectroscopy (EIS) provides insights into the carrier separation efficiency, migration rate, surface reaction activity, and light response capability during the photocatalytic process by analyzing parameters including charge transfer resistance, carrier migration resistance, diffusion impedance, and interface capacitance, thereby achieving a mechanistic characterization of photocatalytic activity. Generally, a smaller arc radius indicates lower impedance, higher charge carrier utilization efficiency, and, consequently, stronger photocatalytic activity [52,53]. Figure 6b displays the Nyquist plots of the prepared samples. Among all the prepared samples, the BiOI/CuWO_4_-10 sample exhibits the smallest arc radius. It indicates that this sample has the lowest charge transfer resistance and the highest photocatalytic activity. This conclusion is completely consistent with that obtained from the photocurrent density measurement.

**Figure 7:**
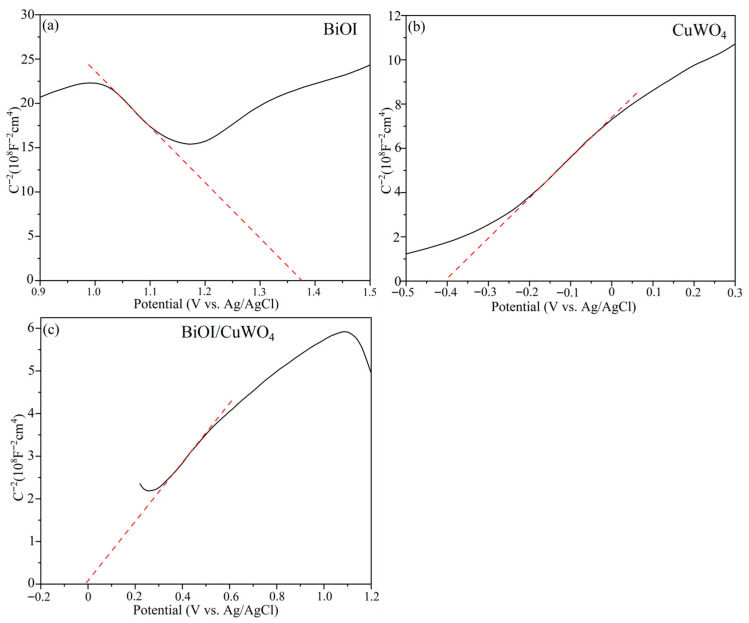
Mott–Schottky curves of the samples: (**a**) BiOI, (**b**) CuWO_4_, and (**c**) BiOI/CuWO_4_.

Figure 7 presents the Mott–Schottky plots of the as-prepared samples. The slopes of the Mott–Schottky plots of BiOI and CuWO_4_ were negative and positive, respectively, suggesting that they were *p*-type and *n*-type semiconductors. As shown in Figure 7a,b, the flat band potentials (E_fb_) of BiOI and CuWO_4_ relative to the Ag/AgCl electrode were −0.63 and 0.09 V, respectively. According to the equation E (NHE) = E (Ag/AgCl) + 0.2, the flat band potential (E_fb_) was calculated to be −0.43 and −0.19 V, respectively, for BiOI and CuWO_4_. Typically, for the *n*-type semiconductor CuWO_4_, the conduction band minimum (CBM) can be calculated using the equation E_CBM_ = E_fb_ + 0.2 [5]. On the other hand, for the *p*-type semiconductor BiOI, the valence band maximum (VBM) can be calculated via the equation E_VBM ≈_ E_fb_ [23]. The VBM and CBM of CuWO4 and BiOI can be calculated using the formula E_VBM_ = E_CBM_ + E_g_, which are the optical bandgap values determined from Figure 5a. On the basis of the data obtained from Figure 5 and Figure 7 and the aforementioned equations, the conduction band minimum and valence band maximum of BiOI can be calculated to be −0.35 eV and +1.37 eV, respectively. Similarly, the conduction band minimum and valence band maximum of CuWO_4_ are +0.01 eV and +2.34 eV, respectively. In addition, the Mott–Schottky plot of BiOI–CuWO4 (Figure 7c) exhibits both positive and negative slopes, indicating the formation of a *p*–*n* heterojunction between the two materials.

**Figure 8:**
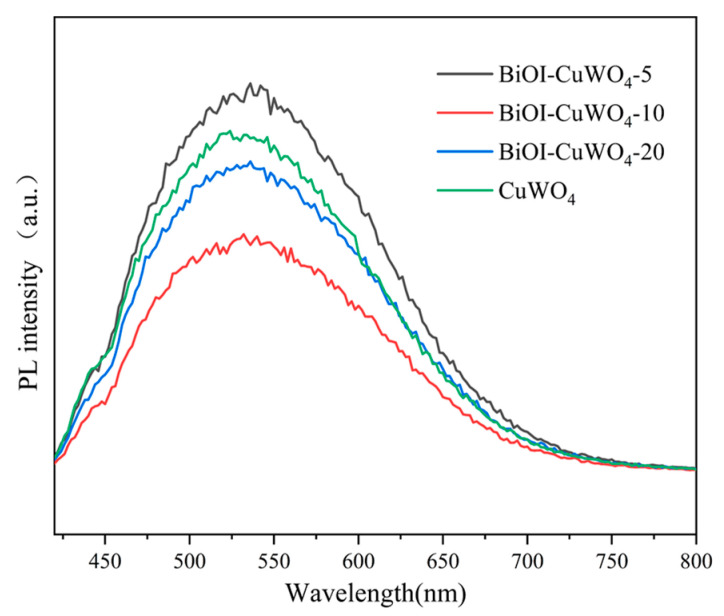
Steady-state PL spectra of the prepared samples.

Photoluminescence (PL) spectroscopy provides insight into photocatalytic activity through features such as fluorescence intensity (reflecting recombination efficiency) and peak position (reflecting energy levels and defects). Typically, lower PL intensity and longer carrier lifetime indicate higher carrier separation efficiency, a lower recombination rate, a greater number of carriers available for photocatalytic reactions, and thus stronger photocatalytic activity [52,53]. Figure 8 shows the steady-state PL spectra of the prepared CuWO_4_ films and BiOI/CuWO_4_ heterojunction films. After coupling with BiOI, the PL intensity of the prepared films decreases to varying degrees, which indicates that the recombination of photogenerated electrons and holes is inhibited. In particular, the BiOI/CuWO_4_-10 sample exhibits the lowest PL intensity, suggesting that this sample possesses the highest photocatalytic activity. This is completely consistent with the conclusion drawn from the results shown in Figure 6.

**Figure 9:**
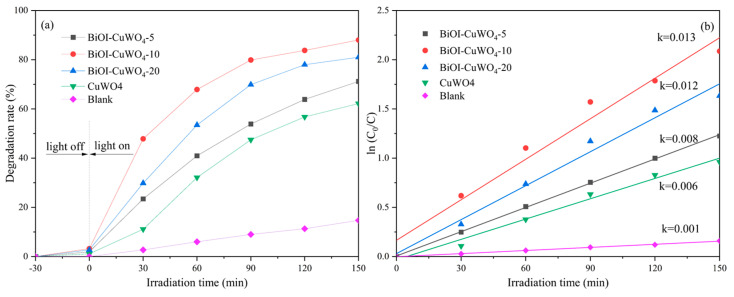
Photocatalytic degradation of MB dye with the prepared CuWO_4_ films and BiOI/CuWO_4_ heterojunction films: (**a**) the concentration of the MB solution as a function of irradiation time, and (**b**) degradation rate curves of the MB solution.

Figure 9 shows the photocatalytic degradation of the organic dye MB by the prepared samples. In Figure 9a, there is some self-degradation of MB dye under irradiation by simulated sunlight. Approximately 15% of MB self-degraded within 150 min. The pristine CuWO_4_ films degraded 60% within the same irradiation time. On the other hand, under the same experimental conditions, the BiOI/CuWO_4_ heterojunction films degraded up to ~88% of the MB. The photocatalytic degradation of MB follows the pseudo-first-order reaction kinetic model shown in Figure 9b. Meanwhile, the degradation rate constant, *k*, of different samples for MB was calculated. The *k* value of the single CuWO_4_ films is 6 × 10^−3^ min^−1^, while the *k* values of the BiOI/CuWO_4_ heterojunction films all increase to a certain extent. Among them, the *k* value of the BiOI/CuWO_4_-10 film is as high as 1.3 × 10^−2^ min^−1^, which is 2.17 times that of the single CuWO_4_ films. Therefore, the modification of BiOI significantly enhances the photocatalytic oxidation activity of CuWO_4_ films.

**Figure 10:**
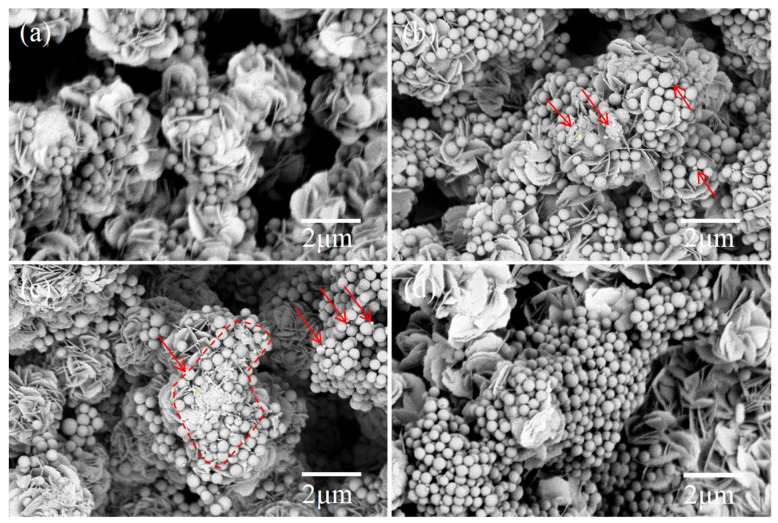
SEM images of the PS nanoparticles that underwent photocatalysis with BiOI/CuWO_4_ films after different irradiation times: (**a**) 40 h, (**b**) 80 h, and (**c**) 120 h, and (**d**) 120 h without light irradiation.

Photocatalytic degradation of PS nanoplastics was also performed. Figure 10 presents the SEM images of PS nanoplastics adsorbed on the surface of the BiOI/CuWO_4_-10 sample after irradiation for a certain period of irradiation time. It can be seen that after 40 h, no significant change was observed (Figure 10a). However, after 80 h, the nanoplastics were significantly reduced in size, and their surfaces became rough and undulating as indicated by the arrows (Figure 10b). After 120 h, the nanoplastics fragmented into small, irregular particles and smaller spheres as indicated by the arrows (Figure 10c). The uneven surfaces and fragmentation of the PS nanoplastics resulted from the destruction of molecular bonds by the oxidative active species generated by the BiOI/CuWO_4_-10 sample. For comparative purposes, we also conducted control experiments under dark conditions, with the results shown in Figure 10d. It can be observed that after 120 h of static storage in the absence of light, the PS nanoparticles remained intact with no signs of reduction in particle size. This indicates that light irradiation is an indispensable condition in the photocatalytic degradation process.

**Figure 11:**
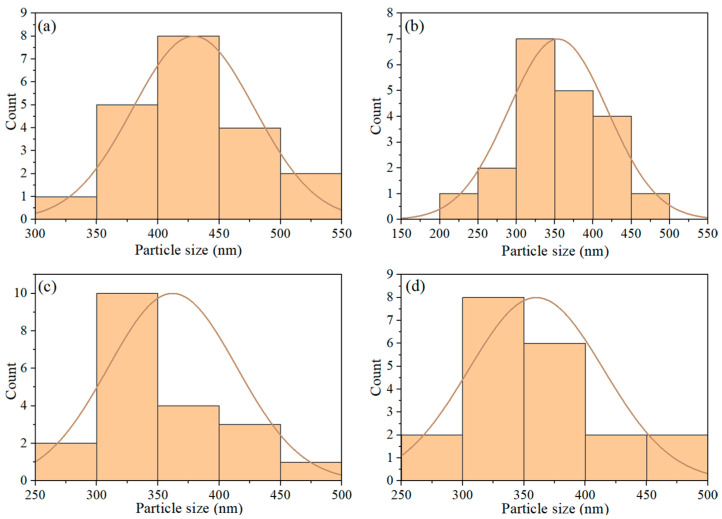
Average particle size of PS nanoplastics during the photocatalytic degradation process: (**a**) 0 h, (**b**) 40 h, (**c**) 80 h, and (**d**) 120 h.

Statistics were collected on the changes in the average particle size of PS nanoplastics during the photocatalytic degradation process, and the results are shown in Figure 11. Before photocatalytic degradation, the average particle size of PS plastics was 425 nm (Figure 11a). After 40 h of irradiation, the average particle size was 350 nm (Figure 11b). After 80 and 120 h, the average particle size further decreased to 325 nm. These results indicate that the heterojunction films possess high photocatalytic activity towards PS nanoplastics.

**Figure 12:**
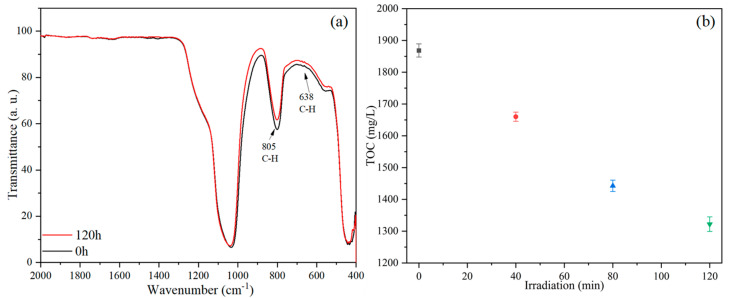
(**a**) FTIR spectra and (**b**) TOC of the reaction solution before and after photocatalytic degradation.

Figure 12a shows the FTIR spectra of PS nanoplastics before and after photocatalytic degradation. After 120 h of light irradiation, the characteristic peaks in the FTIR spectrum of PS change significantly. The peak at 805 cm^−1^, corresponding to the out-of-plane bending vibration of C–H in monosubstituted benzene rings, and the peak at 638 cm^−1^, assigned to the deformation vibration of C–H bonds, both show a tendency of intensity attenuation. This may be caused by the dehydrogenation or oxidation reactions of C–H bonds in aromatic rings and aliphatic chains [54,55]. Figure 12b shows the evolution of total organic carbon (TOC) in the solution at different stages of the photocatalytic evaluation. It can be observed that with prolonged irradiation time, the TOC of the solution continuously decreased, dropping from an initial value of 1868 mg/L to a value of 1322 mg/L after 120 h. This indicates that the TOC in the solution progressively decreased as irradiation time increased. Since the only source of TOC in the solution is the PS nanoplastics, this result demonstrates a continuous reduction in the amount of PS nanoplastics present, i.e., complete mineralization into inorganic carbon species such as carbon dioxide.

**Figure 13:**
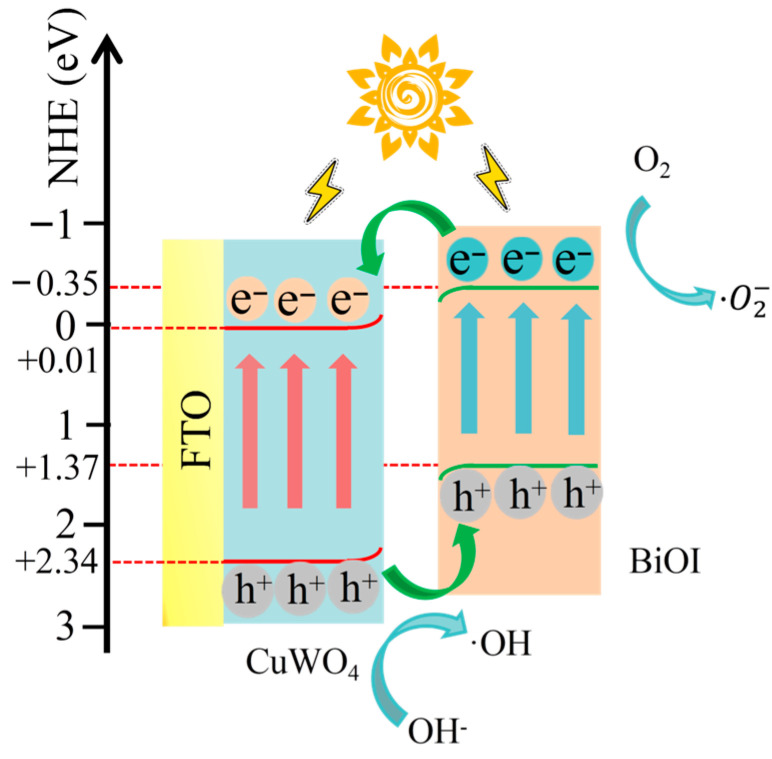
Possible mechanism for the enhancement of CuWO_4_ photocatalytic activity by BiOI modification.

After the coupling of BiOI and CuWO_4_, the UV–visible diffuse reflectance spectra of the heterojunction in Figure 5 show that the absorption edge shifts from 530 nm of CuWO_4_ to above 660 nm, and the absorption intensity in the range of 300–800 nm is significantly enhanced. This means that the heterojunction can capture more UV–visible–NIR light photons and generate more photogenerated carriers, providing sufficient reactive species (such as ·OH,·O_2−_, etc.) for catalytic reactions. According to the results presented in Figure 5 and Figure 7, the conduction band minimum (CBM) of BiOI is −0.35 eV (vs. NHE), and the valence band maximum (VBM) is +1.37 eV; the CBM of CuWO_4_ is +0.01 eV, and the VBM is +2.34 eV. The energy band structures of BiOI and CuWO_4_ have a natural matching property, usually forming a Type II heterojunction: the CBM position of BiOI is more negative than that of CuWO_4_, while the VBM position of CuWO_4_ is more positive than that of BiOI (Figure 13). When the heterojunction is excited by light, both BiOI and CuWO_4_ will generate electrons and holes. A portion of the photogenerated electrons in the conduction band of BiOI will transfer from the conduction band of CuWO_4_, while the remaining photogenerated electrons combine with oxygen molecules in the environment to produce superoxide anion radicals ($\cdot O_{2}^{-}$), since the redox potential of O_2_/$\cdot O_{2}^{-}$ is −0.33 V vs. NHE [56]. Our existing research findings have demonstrated that superoxide anion radicals ($\cdot O_{2}^{-}$) serve as the key reactive oxygen species in the BiOI/CuWO_4_ heterojunction system [37]. On the other hand, some of the photogenerated holes in the valence band of CuWO_4_ will migrate to the valence band of BiOI, while the remaining photogenerated holes react with hydroxide ions (OH^−^) to generate hydroxyl radicals (OH) because the redox potential of OH^−^/OH is +2.27 V vs. NHE [56]. This electron–hole cross-interface separation makes the photogenerated electrons concentrate in the CB of CuWO_4_, and the photogenerated holes concentrate in the VB of BiOI, completely avoiding the recombination of photogenerated electrons and holes in a single semiconductor. This mechanism is verified by PL spectroscopy (Figure 8) and photocurrent tests (Figure 6). Specifically, the PL peak intensity of pure CuWO_4_ is relatively high, while that of the BiOI/CuWO_4_ heterojunction is significantly reduced, which proves that the carrier recombination rate is greatly decreased. Meanwhile, the transient photocurrent test shows that the heterojunction has a higher photocurrent density, further confirming the improvement in carrier separation efficiency.

## 4. Conclusions

In this study, BiOI-modified CuWO_4_ composite thin films were prepared on FTO glass substrates using a combination of the hydrothermal method and the SILAR method. Microstructural characterization confirmed the formation of BiOI/CuWO_4_ heterojunctions. The absorption threshold of pure CuWO_4_ is 530 nm, while that of the BiOI/CuWO_4_ heterojunction films extends to 660 nm. Moreover, the latter shows significantly improved light absorption capacity across the entire solar spectrum. The results of photoelectric tests indicate that the photocurrent density increased by up to 4.5 times, and the transfer resistance of photogenerated carriers is also lower. The heterojunction formation has significantly enhanced the photocatalytic oxidation capacity of CuWO_4_. In the photocatalytic degradation of the organic dye MB, the activity of the BiOI/CuWO_4_-10 sample is 2.17 times that of the individual CuWO_4_ films. Meanwhile, the sample also exhibits certain activity in the photocatalytic degradation of PS nanoplastics, capable of reducing the average particle size of nanoplastics from 425 nm to 325 nm within 80 h.

## Data Availability

Authors confirm that all relevant data supporting this article are included in the article. Other necessary data will be made available on request.
